# A retrospective comparison of albumin versus mannitol priming fluid with relation to postoperative atrial fibrillation

**DOI:** 10.1111/jocs.16960

**Published:** 2022-09-18

**Authors:** M. Scott Binder, YingXing Wu, Joseph W. Baker, Joseph F. Rowe, David A. Wyatt, Cynthia Choate, Steven Poelzing, Mark Joseph

**Affiliations:** ^1^ Departments of Cardiology, Cardiothoracic Surgery Virginia Tech Carilion Roanoke Virginia USA; ^2^ Department of Health Analytics Virginia Tech Carilion Roanoke Virginia USA; ^3^ Department of Biomedical Engineering and Mechanics Virginia Tech Fralin Biomedical Research Institute Roanoke Virginia USA

**Keywords:** albumin, perinexus, postoperative atrial fibrillation, priming fluid

## Abstract

**Background and Aim of the Study:**

Postoperative atrial fibrillation (POAF) is a common complication following cardiac surgery which can result in increased mortality and increased healthcare costs. During Hurricane Maria (2017), a nationwide shortage of mannitol occurred, and our institution switched to the utilization of albumin as a priming fluid solution. We observed decreased rates of POAF during that time and began alternating albumin and mannitol priming fluid solutions. We hypothesized this observation may be from altered perinexal conduction from albumin utilization.

**Methods:**

A retrospective chart review of all patients from January 2020 through December 2020 who underwent cardiac surgery was performed, to determine if albumin was associated with reduced POAF rates. Two hundred and thirteen patients were identified and 4 were excluded. Two hundred and nine patients (110 albumin priming fluid and 99 mannitol priming fluid) were included in our final analysis.

**Results:**

Analysis was performed for all patients with POAF and in patients with new‐onset AF (without a history of prior AF) after surgery. POAF rates showed no statistically significant difference between cohorts. For all patients, POAF occurred in 43% of the albumin subgroup and 47% of the mannitol subgroup (*p* = .53) and for patients with new‐onset AF, POAF occurred in 35% of the albumin subgroup versus 42% of the mannitol subgroup (*p* = .36). Logistic regression revealed that age, ejection fraction and cardiopulmonary bypass time was associated with POAF, in our cohort.

**Conclusions:**

The use of albumin compared to mannitol as priming fluid solutions was not associated with statistically significant reductions in POAF rate, in our population.

## 1 INTRODUCTION

Postoperative atrial fibrillation (POAF) is a common complication following cardiac surgery, occurring in 20%–50% of cases.^1^, ^2^ POAF will typically peak between postoperative Days 2 and 4 but 90%–98% will resolve within 4–6 weeks.^2^, ^3^ Incidence will vary based upon the type of surgery as well as modifiable (hypertension, obesity, and tobacco use) and non‐modifiable (Caucasian ancestry, male sex, and older age) risk factors.^3^, ^4^ POAF has been associated with increased mortality, increased hospital length of stay, and healthcare costs.^5^, ^6^

During hurricane Maria (2017), a nationwide shortage of mannitol occurred, and our institution switched to utilizing albumin as part of our priming fluid solution. A decrease in POAF rates was observed, so our institution started to alternate albumin and mannitol priming fluid solutions at that time. We hypothesized that altered perinexal conduction, the region adjacent to gap junctions, may explain the reduced observed POAF rates.^7^, ^8^ In vitro studies have revealed decreased interstitial edema and improved ephaptic coupling with albumin solutions compared to mannitol.^9^, ^10^ The mechanism is presumed to be through interactions of albumin with the endothelial glycocalyx, resulting in decreased vascular permeability.^11^, ^12^ We performed a retrospective cohort study of all patients who underwent cardiac surgery at our institution from January 2020 through December 2020, to determine if albumin priming fluid solution could help reduce POAF rates.

## 2 MATERIALS AND METHODS

A retrospective cohort study was initiated using deidentified patient data from January 2020 through December 2020 and identified 213 patients who underwent cardiac surgery at our institution. Given this was a retrospective study using deidentified data, Institutional Review Board (IRB) waiver was granted (IRB‐21‐1504). A consent statement and clinical trial registration were not applicable for our study, given the retrospective nature. Four patients were excluded from analysis given incomplete chart data, repeat cardiac surgery, or death during the procedure. POAF was defined using the American Association for Thoracic Surgery (AATS) clinical definition of intra‐ or postoperative AF requiring treatment or anticoagulation and/or extending the duration of hospitalization.^3^ Preoperative cardiac medications, including beta blockers and blood pressure medications, were continued postoperatively. In addition, for elective procedures, our current protocol includes perioperative dose of beta blocker and postoperative resumption once weaned from inotropic medication with prophylactic dose of amiodarone given to all patients, unless preoperative QTC is >500 ms or patient has known bradycardia or heart block. Antiplatelet agents, including clopidogrel, ticagrelor, and prasugrel, were held to allow for washout unless the surgery was emergent in addition to ACE inhibitors and ARBs for 48–72 h.

The mannitol prime solution consisted of 50 g of mannitol in 1 L of lactated ringer (LR) with additional bicarb and heparin for a total volume of 1310 ml and the albumin prime solution consisted of 50 g of 25% Albumin with bicarb and heparin in 1 L LR for the total volume of 1260.

Continuous variables were presented as mean ± standard deviation or median (interquartile range) and categorical variables as percentage. *T*‐tests or Mann–Whitney *U*‐tests and chi‐squared tests were used for between‐groups comparison accordingly. Logistic Regressions were used to evaluate the effect of type of priming fluid and other variables on POAF. Local regressions were used to fit smooth curves between new‐onset POAF and other predictors. Separate analyses were conducted for all patients with POAF and for patients with new‐onset AF after surgery without a prior history of AF. Statistical analyses were performed using R4.0.

## 3 RESULTS

Two hundred and nine patients (mean age 67 ± 11 years, 70% male, 96% Caucasian ancestry) were included in the final analysis, with 110 having received albumin and 99 having received mannitol solutions. Patients were well balanced in their baseline characteristics, with the notable exception of higher rates of diabetes and current tobacco use in the mannitol cohort (Table 1). Medication use at discharge was also similar between groups, apart from a slightly higher rate of apixaban use in the albumin cohort (Table 2).

**Table 1:** Baseline demographics of patients in the albumin and mannitol cohorts

	All patients	No history of atrial fibrillation			Patients with or without history of atrial fibrillation		
		Albumin	Mannitol	*p* value	Albumin	Mannitol	*p* value
Number of patients	209	86	83		110	99	
Age	67 ± 11	66 ± 11	66 ± 11	.95	67 ± 11	67 ± 11	.67
Male	70.5%	74.4%	71.1%	.63	68.2%	72.7%	.47
Ethnicity				.42			.41
White	96.6%	96.5%	95.2%		97.3%	96.0%	
African American	2.9%	2.3%	4.8%		1.8%	4.0%	
Nonwhite Hispanic	0.9%	1.2%			.9%		
Body mass index	30 ± 6	30 ± 6	30 ± 6	.86	30 ± 6	30 ± 6	.96
Ejection fraction (%)	58 ± 10	59 ± 7	58 ± 10	.17	58 ± 10	57 ± 10	.61
Smoking				**.05**			**.04**
Current	**10.4%**	**7.0%**	**18.1%**		**5.5%**	**15.2%**	
Prior	46%	44.2%	45.8%		45.5%	46.5%	
Never	43.8%	48.8%	36.1%		49.1%	38.4%	
Ischemic cardiomyopathy	74.4%	73.3%	79.5%	.34	70.0%	78.8%	.15
Hypertension	81.5%	83%	83%	.92	82%	81%	.85
Diabetes	36%	33%	46%	.08	**29%**	**43%**	**.03**
Hyperlipidemia	74.5%	76%	76%	.96	75%	74%	.89
History of cardiac surgery	3.5%	6%	1%	.21	5%	2%	.29
Procedure type				.37			.28
CABG (+Other)	61.5%	62%	71%		55%	68%	
Valve replacement/repair (+Other)	19.5%	19%	16%		23%	16%	
CABG + Valve (+Other)	10.5%	8%	8%		11%	10%	
Other (includes aortic root/ascending aorta repair/replacement, SVC repair, LVAD implantation, and pericardiectomy)	8.5%	12%	5%		11%	6%	
Operative status				.37			.25
Elective	95.5%	95.1%	97.5%		94.2%	96.8%	
Urgent	3.1%	2.5%	2.5%		2.9%	3.2%	
Emergent	2.9%	2.5%			2.9%		
Cardioplegia route				.36			.50
Anterograde	44.5%	43%	46%		43%	46%	
Retrograde	5%	7%	2%		7%	3%	
Dual	50.5%	49%	51%		50%	51%	
CPB time (h)	1.7 (1.3, 2.1)	1.6 (1.2, 2.3)	1.7 (1.2, 2.1)	.73	1.7 (1.2, 2.4)	1.7 (1.3, 2.1)	.48
Cross‐clamp time (min)	69 (44, 97)	63 (43, 98)	69 (46, 96)	.66	66 (44, 97)	72 (46, 99)	.65
Hospital death	3%	2%	1%	.99	5%	1%	.12

*Note*: Statistically significant differences (defined as *p* ≤ .05) are identified in bold.

**Table 2:** Medication use in patients after cardiac surgery

	No history of atrial fibrillation		Patients with or without history of atrial fibrillation	
	Albumin	Mannitol	Albumin	Mannitol
Lisinopril	5%	7%	5%	7%
Metoprolol	79%	82%	77%	83%
Carvedilol	7%	4%	8%	3%
Losartan	5%	5%	5%	5%
Valsartan	0%	1%	1%	1%
Furosemide	72%	76%	75%	77%
Bumetanide	8%	1%	8%	1%
Torsemide	1%	0%	1%	0%
Hydrochlorothiazide	0%	1%	0%	1%
Atorvastatin	51%	60%	47%	59%
Pravastatin	3%	0%	5%	0%
Simvastatin	1%	1%	3%	1%
Rosuvastatin	24%	29%	26%	29%
Aspirin	100%	100%	100%	100%
**Apixaban**	**9%**	**7%**	**16%**	**10%**

*Note*: Statistically significant differences (defined as *p* ≤ .05) are identified in bold.

**Figure 1:**
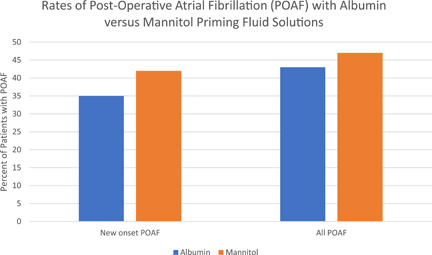
Rates of POAF in all patients and in patients with new‐onset AF, following cardiac surgery. No significant differences were found between priming fluid solutions. POAF, postoperative atrial fibrillation.

POAF rates showed no statistically significant difference between cohorts. In all patients, POAF occurred in 43% of the albumin subgroup compared with 47% in the mannitol subgroup (*p* = .53). In patients with new‐onset AF after surgery, POAF occurred in 35% of the albumin subgroup compared with 42% of the mannitol subgroup (*p* = .36, Figure 1).

**Figure 2:**
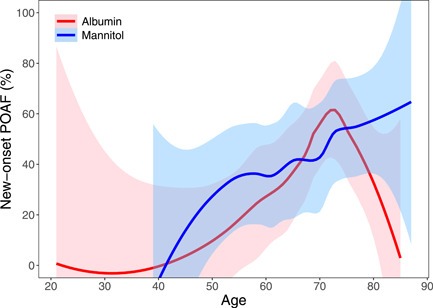
Local regression of new‐onset POAF versus age. The red line indicates albumin and the blue line indicates mannitol. Shaded areas represent 95% confidence bands using a global variance estimate. POAF, postoperative atrial fibrillation.

**Figure 3:**
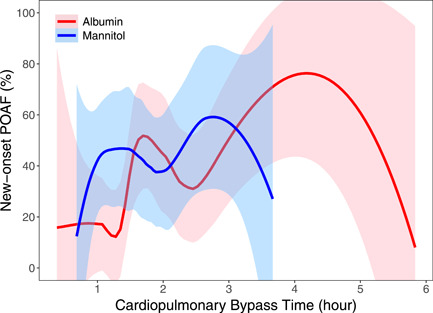
Local regression of new‐onset POAF versus cardiopulmonary bypass time, in hours. The red line indicates albumin and the blue line indicates mannitol. Shaded areas represent 95% confidence bands using a global variance estimate. POAF, postoperative atrial fibrillation.

**Figure 4:**
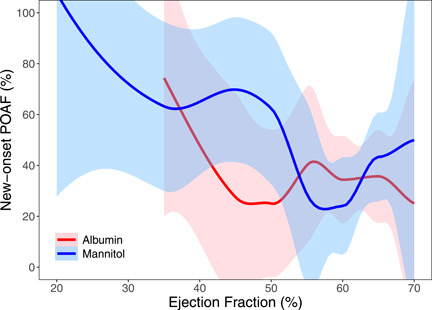
Local regression of new‐onset POAF versus ejection fraction. The red line indicates albumin and the blue line indicates mannitol. Shaded areas represent 95% confidence bands using a global variance estimate. POAF, postoperative atrial fibrillation.

Logistic regression found that POAF significantly increased with age and cardiopulmonary bypass (CPB) time and that POAF significantly decreased with an increase in ejection fraction (Tables 3 and 4, Figures 2, 3, 4). Albumin may show more benefit for certain subgroups, including more elderly patients and patients with mid‐range ejection fraction (40%–50%), but further study is needed to clarify the significance of these findings.

**Table 3:** Logistic regression for all patients with POAF showing significant risk modifiers for POAF in our patient cohort

	Odds ratio (95% CI)	*p* value
Age (per 10 years)	1.78 (1.30, 2.43)	<0.01
Ejection fraction (per 10%)	0.63 (0.45, 0.88)	<0.01
CPB time (per hour)	1.66 (1.11, 2.47)	0.01

Abbreviations: CI, confidence interval; CPB, cardiopulmonary bypass; POAF, postoperative atrial fibrillation.

**Table 4:** Logistic regression for patients with new‐onset POAF showing significant risk modifiers for POAF in our patient cohort

	Odds ratio (95% CI)	*p* value
Age (per 10 years)	1.59 (1.13, 2.22)	<0.01
Ejection fraction (per 10%)	0.65 (0.45, 0.95)	0.02
CPB time (per hour)	1.65 (1.08, 2.52)	0.02

Abbreviations: CI, confidence interval; CPB, cardiopulmonary bypass; POAF, postoperative atrial fibrillation.

## 4 DISCUSSION

It is routine practice to prepare CPB circuits with a priming solution before the initiation of open‐heart procedures. Priming solutions are essential to the preparation of a CPB machine and exert physiological effects in patients undergoing open heart procedures. Historically, albumin has been a common additive to priming solutions to maintain colloid oncotic pressure (COP) and reduce edema, while mannitol was favored to reduce postoperative renal dysfunction and act as a free radical scavenger.^13^, ^14^

Priming fluid has been studied previously in cardiac surgery with respect to pediatric patients in regard to the risk of bleeding with use of various types of priming fluid.^15^, ^16^ Bleeding and transfusion requirements have been shown to be independent risk factors for POAF.^17^ This is thought to be secondary to the increased inflammatory response of blood in the pericardium being a nidus for POAF. Although current studies show differences in using crystalloid and colloid‐based fluids, no current study compares the use of albumin to that of mannitol, both routinely used in cardiac operating rooms frequently. Albumin priming has been shown previously to better preserve platelet function, COP, and fluid balance with weight gains after cardiac surgery, compared to crystalloids, but the clinical significance of these findings has not been elucidated.^18^

Our study found no significantly reduced rates of POAF with utilization of an albumin priming fluid solution when compared to mannitol priming fluid solution. A prior prospective trial of 100 patients in 1989 by Marelli et al. showed no significant difference with the addition of 50 g of albumin to LRs solution, with additional costs incurred.^19^ Since that time, however, studies have shown the damaging effects of CPB on the endothelial glycocalyx, resulting in increased tissue permeability and edema.^20^, ^21^ Retrospective studies have since shown improved mortality and less positive fluid balance with albumin use compared to crystalloids in cardiac surgery.^22^, ^23^ A single‐center prospective clinical trial (NCT02560519) was recently published by Pesonen et al., and found no significant difference in adverse events, including arrhythmias (defined as ventricular arrhythmias, POAF, or need for a permanent pacemaker) with use of albumin compared to ringer acetate after cardiac surgery.^24^

While perinexal conduction was altered with in‐vitro studies of albumin versus mannitol solutions, human metabolism, and interstitial fluid exhibit much greater regulation. Infused albumin typically has a half‐life of around 110 min in healthy subjects.^25^ Catabolism of albumin may be increased by glycosylation, which is known to occur in 2‐ to 3‐fold increases in patients with diabetes.^26^ Given that most episodes of POAF occur between 2 and 4 days, priming fluid volume containing mannitol or albumin may not be sufficient to cause significant changes in glycocalyx integrity, but further study is required.

Our study has several strengths, including the large size and prevalent POAF rates. POAF rates in our cohort were generally at the upper limit of prior studies (around 40%–45%), which we hypothesize was due to the large proportion of male patients who were of Caucasian ancestry, both nonmodifiable risk factors for POAF.^6^ Our groups were also very well matched which allows for the limitation of confounding factors.

Limitations of our study include the single‐center retrospective design, which may increase selection bias. Generalizability of our results may also be limited due to lack of a diverse population at our institution. Another limitation is that looking at priming solutions is extremely variable, with practice patterns differing greatly by region and even within a practice.^27^ For example, we employ the use of retrograde priming using the patient's blood, which is helpful to avoid hemodilution but also means that the amount of priming fluid each patient receives can be varied. Additionally, multiple risk factors exist for POAF. Left atrial enlargement has been associated with POAF following CABG and aortic valve replacements.^28^, ^29^ Smoking, which was increased in the mannitol group, has not been associated with POAF, while diabetes, also increased in the mannitol group, has been associated with increased POAF rates.^30^, ^31^ While our study could not control for all possible risk enhancers, further research is needed into the highest‐risk subgroups to determine if priming fluid could alter POAF rates in certain high‐risk populations.

POAF is likely secondary to multiple variables, of which isolating individual variables can be very difficult. We believe further studies may identify subgroups at higher risk who may see more of a benefit from altered perinexal conduction.

## 5 CONCLUSION

Utilization of albumin versus mannitol priming fluid solution was not associated with statistically significant decreases in the rates of POAF.

## AUTHOR CONTRIBUTIONS

**M. Scott Binder**: Concept/design; data analysis/interpretation; drafting article; data collection; approval of article. **YingXing Wu**: Data analysis/interpretation; statistics; critical revision of article; approval of article. **Joseph W. Baker**: Concept/design; critical revision of article; approval of article. **Joseph F. Rowe**: Concept/design; critical revision of article; approval of article. **David A. Wyatt**: Concept/design; critical revision of article; approval of article. **Cynthia Choate**: Concept/design; critical revision of article; approval of article. **Steven Poelzing**: Concept/design; data analysis/interpretation; critical revision of article; approval of article. **Mark Joseph**: Concept/design; critical revision of article; approval of article.

## CONFLICT OF INTEREST

The authors declare no conflict of interest.
